# Opposed Effects of Dityrosine Formation in Soluble and Aggregated α-Synuclein on Fibril Growth

**DOI:** 10.1016/j.jmb.2017.09.005

**Published:** 2017-10-13

**Authors:** Michael M. Wördehoff, Hamed Shaykhalishahi, Luca Groß, Lothar Gremer, Matthias Stoldt, Alexander K. Buell, Dieter Willbold, Wolfgang Hoyer

**Affiliations:** 1 Institut für Physikalische Biologie, Heinrich-Heine-Universität Düsseldorf, 40204 Düsseldorf, Germany; 2 Institute of Complex Systems (ICS-6), Structural Biochemistry, Research Centre Jülich, 52425 Jülich, Germany

**Keywords:** DiY, dityrosine, α-syn, α-synuclein, ThT, thioflavin T, IDP, intrinsically disordered protein, PD, Parkinson's disease, OS, oxidative stress, ROS, reactive oxygen species, WT, wild type, Y-to-A, tyrosine to alanine (mutants), UV, ultraviolet (light), SEC, size exclusion chromatography, AFM, atomic force microscopy, SDS-PAGE, sodium dodecyl sulfate polyacrylamide gel electrophoresis, protein aggregation, amyloid, Parkinson's disease, oxidative stress, intrinsically disordered proteins

## Abstract

Parkinson's disease is the second most common neurodegenerative disease. It is characterized by aggregation of the protein α-synuclein (α-syn) in Lewy bodies, mitochondrial dysfunction, and increased oxidative stress in the substantia nigra. Oxidative stress leads to several modifications of biomolecules including dityrosine (DiY) crosslinking in proteins, which has recently been detected in α-syn in Lewy bodies from Parkinson's disease patients. Here we report that α-syn is highly susceptible to ultraviolet-induced DiY formation. We investigated DiY formation of α-syn and nine tyrosine-to-alanine mutants and monitored its effect on α-syn fibril formation *in vitro*. Ultraviolet irradiation of intrinsically disordered α-syn generates DiY-modified monomers and dimers, which inhibit fibril formation of unmodified α-syn by interfering with fibril elongation. The inhibition depends on both the DiY group and its integration into α-syn. When preformed α-syn fibrils are crosslinked by DiY formation, they gain increased resistance to denaturation. DiY-stabilized α-syn fibrils retain their high seeding efficiency even after being exposed to denaturant concentrations that completely depolymerize non-crosslinked seeds. Oxidative stress-associated DiY crosslinking of α-syn therefore entails two opposing effects: (i) inhibition of aggregation by DiY-modified monomers and dimers, and (ii) stabilization of fibrillar aggregates against potential degradation mechanisms, which can lead to promotion of aggregation, especially in the presence of secondary nucleation.

## Highlights

• Oxidative stress can lead to dityrosine (DiY) crosslinks in α-synuclein (α-syn).
• α-Syn is highly susceptible to DiY formation by UV light irradiation.
• DiY-crosslinked soluble α-syn inhibits aggregation of unmodified α-syn.
• DiY crosslinking of α-syn fibrils stabilizes on-pathway aggregation seeds.
• DiY formation in α-syn has opposed effects on the pathogenic aggregation process.

## Graphical abstract

**Figure:**
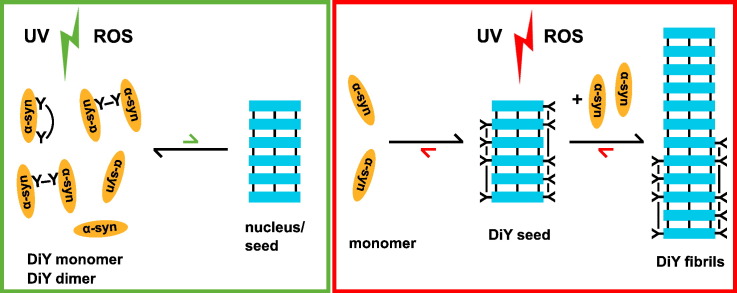

## Introduction

Parkinson's disease (PD) is the second most common neurodegenerative disease with a prevalence of around 1% in people older than 65 years [1], resulting in approximately 100,000 deaths in 2013 [2]. The lifetime risk to develop PD is around 2% for men and 1.3% for women with a tendency to increase in an aging society [3]. On the molecular level, PD pathophysiology shows abnormal aggregation of the intrinsically disordered protein (IDP) α-synuclein (α-syn) [4], as well as increased oxidative stress (OS), especially in dopaminergic neurons of the substantia nigra [5].

Aggregation of α-syn into amyloid fibrils and finally intracellular Lewy body deposits is a central feature of PD and other Lewy body diseases [4]. This is reflected in the genetics of familial forms of PD, where multiplication of or point mutations (A53T, A30P, E46K, G51D) in the *SNCA* gene (encoding α-syn) cause hereditary early-onset forms of PD [6]. Aggregation of α-syn is detrimental to cells in many ways as it decreases the free α-syn monomer pool, thereby disturbing the physiological function of α-syn, that is, maintenance of the synaptic vesicle pool [7], [8] and dopamine trafficking and homeostasis [9]. Moreover, various aggregated species of α-syn have been shown to form pores in the cell membrane [10], damage mitochondria [11], destabilize microtubules [12], [13], and cause endoplasmatic reticulum stress [14].

Besides α-syn aggregation, OS is a central hallmark of PD [5]. The substantia nigra is a brain region with increased OS. First, its high dopamine content is a source of reactive oxygen species (ROS), as the deamination of dopamine by monoamine oxidase generates H_2_O_2_, which is a strong oxidizing agent [15]. Besides that, dopamine autooxidates to dopamine *o*-quinone and aminochrome, thereby creating toxic superoxide and hydroxyl radicals [16]. Moreover, dopaminergic neurons contain high amounts of iron, which is known to catalyze ROS production via the Fenton reaction [17]. Also, mitochondrial dysfunction, for example, inhibition of complex I, is part of PD pathophysiology [18]. Complex I inhibition results in increased production of ROS [19]. This can, for example, be triggered by 1-methyl-4-phenyl-1,2,3,6-tetrahydropyridine, a drug that inhibits complex I and thereby leads to characteristic PD symptoms, making it an animal model for PD [20]. Besides that, familial early-onset forms of PD are caused by mutations in *PINK1* [21], *PRKN* [22], and *PARK7* [23], which code for proteins that usually associate with mitochondria in OS conditions and either prevent oxidative damage [24] or initialize autophagy of already damaged mitochondria [25].

OS is harmful to cells in many ways, as it leads to lipid peroxidation, DNA damage, and dityrosine (DiY) formation in proteins [26]. DiY crosslinks are formed when two tyrosyl radicals react with each other to form a C_ortho_–C_ortho_ covalent bond between the two phenol moieties [27]. Elevated DiY levels have been shown to exist in the brains of Alzheimer's disease patients [28], and DiY crosslinking of amyloid-β increases its toxicity by enhanced aggregation propensity and stabilization of already aggregated amyloid-β fibrils [29]. In PD, DiY and α-syn are colocalized in Lewy bodies of substantia nigra brain sections [30]. Moreover, DiY is present in ROS-induced α-syn aggregates in dopaminergic SH-SY5Y cells [31] and is a marker of OS in a 1-methyl-4-phenyl-1,2,3,6-tetrahydropyridine model of PD [32]. DiY crosslinking of α-syn *in vitro* has been performed by incubating α-syn with peroxynitrite/CO_2_ [33], [34], [35], CuCl_2_/H_2_O_2_ [30], [34], and cytochrome c/H_2_O_2_ [36]; by prolonged incubation [37]; or upon purification [38]. To specifically achieve DiY modification of α-syn, photosensitization of tris(bipyridine)-ruthenium(II) chloride in presence of ammonium persulfate was performed, demonstrating toxicity of the photoinduced oligomeric species to SH-SY5Y cells [39]. The *in vitro* studies have consistently linked DiY formation to stabilization of preformed aggregates [33], [36], [37], [39]. In contrast, the reported effects on α-syn fibril formation varied, comprising complete inhibition of fibril formation [34], reduction of the fibril amount [39], formation of off-pathway aggregates [36], and increased fibril formation [30].

In previous *in vitro* studies, extensive crosslinking typically resulted in α-syn multimers up to high-n oligomers [30], [33], [34], [36], [39]. An alternative method for DiY generation is direct photolysis by ultraviolet (UV) irradiation at a wavelength of around 280 nm, which generates tyrosyl radicals by photo-ejection of electrons [40]. DiY formation can in this case be directly followed by an increasing fluorescence emission at 410 nm, as has been applied in studies of the DiY formation characteristics of, for example, calmodulin [41] and insulin [42].

Here we show that α-syn is highly susceptible to DiY formation by UV irradiation, resulting in DiY-modified α-syn monomers and dimers but not higher-n oligomers. We compare UV-induced DiY formation of wild-type (WT) α-syn and several tyrosine-to-alanine (Y-to-A) mutants. We investigate the consequences of DiY formation for α-syn fibril formation and observe both inhibitory and stabilizing effects, depending on whether soluble or aggregated α-syn is DiY-modified.

## Results

### α-Syn is highly susceptible to DiY formation upon UV irradiation at 280-nm wavelength

**Fig. 1:**
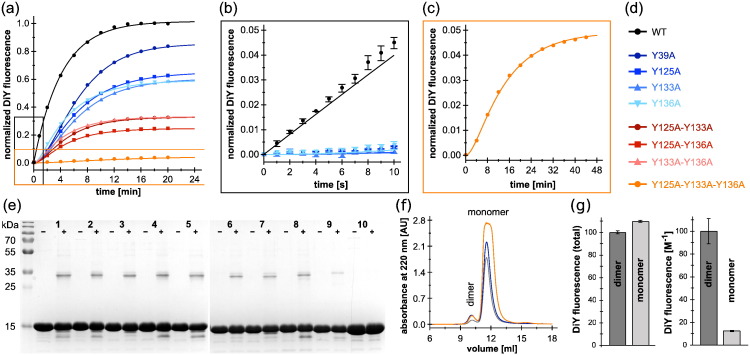
DiY formation kinetics of WT α-syn and Y-to-A mutants. (a) DiY formation kinetics of WT α-syn and single/double/triple Y-to-A mutants upon UV irradiation at 280 nm wavelength, monitored by DiY fluorescence at 410 nm. Fluorescence intensities were normalized by setting the highest fluorescence value of WT DiY-α-syn to unity. All curves are means of triplicate measurements. Solid lines represent fits to a consecutive two-step reaction, except for WT α-syn, for which a one-step first-order model was applied. (b) Zoom into the first 10 s of the kinetics of WT α-syn and single Y-to-A mutants. (c) Zoom into the DiY formation kinetics of the triple Y-to-A mutant, Y125A–Y133A–Y136A. (d) α-Syn variants investigated in this study. (e) SDS-PAGE of WT α-syn and Y-to-A mutants before (−) and after (+, samples taken when the DiY fluorescence plateau was reached) UV irradiation. 1: WT α-syn, 2–9: single/double/triple Y-to-A mutants in same order as in panel d, 10: quadruple Y-to-A tyrosine knockout mutant. (f) SEC of WT DiY-α-syn, C-term DiY-α-syn (Y39A), and N-term DiY-α-syn (Y125A–Y133A–Y136A). (g) DiY fluorescence in dimer and monomer peaks after SEC of WT DiY-α-syn, total fluorescence, and fluorescence intensity normalized to protein concentration are shown as means of triplicates.

To study the effect of DiY crosslinks on α-syn aggregation, we generated DiY-modified α-syn by UV irradiation. α-Syn was expressed and purified with an N-terminal acetyl group, corresponding to the physiological state [43]. We chose UV-irradiation-induced DiY formation, as it allows online monitoring of DiY formation kinetics by fluorescence spectroscopy and enables preparation of sufficient amounts of DiY-crosslinked protein for biophysical studies. As there are no tryptophans and cysteines in α-syn and only two phenylalanines (*λ*_Ex (max)_: 258 nm), its four tyrosine residues are the major target for photo-oxidative modifications [42]. We therefore applied UV irradiation at 280 nm, using the xenon lamp of a spectrofluorometer as the light source, to selectively excite tyrosine and transform it into a radical, which leads to the formation of DiY, its most abundant photo-oxidation product [44]. The kinetics of DiY formation were simultaneously recorded by monitoring DiY fluorescence emission at 410 nm. WT α-syn rapidly formed DiY upon irradiation in the spectrofluorometer, reaching a DiY fluorescence intensity plateau within minutes (Fig. 1a, black trace, Supplementary Fig. S1). The set of α-syn species resulting from UV-induced DiY formation was analyzed by size exclusion chromatography (SEC) and sodium dodecyl sulfate polyacrylamide gel electrophoresis (SDS-PAGE) (Fig. 1e, f). Monomers and dimers but no higher-n oligomers were detected. In SDS-PAGE, weak additional bands appeared below the monomer band upon UV irradiation, which presumably correspond to tyrosine-dependent fragmentation products (Supplementary Fig. S2) [45], [46].

In order to test if the four tyrosine residues in α-syn possess different propensities for DiY formation, we created nine Y-to-A mutants of α-syn, the single mutants Y39A, Y125A, Y133A, and Y136A; double mutants Y125A–Y133A, Y125A–Y136A, and Y133A–Y136A; the triple mutant Y125A–Y133A–Y136A; and a quadruple tyrosine knockout mutant. All single Y-to-A mutants exhibited similar DiY formation kinetics, with Y39A reaching a slightly higher final fluorescence intensity compared to the other single mutants (Fig. 1a, b, blue traces). Similarly, the three double mutants showed comparable DiY formation kinetics (Fig. 1a, b, red traces). This indicates that the DiY formation propensity does not differ greatly between the four tyrosines in α-syn. The extent of DiY formation as judged from the final DiY fluorescence intensities increased with the number of tyrosine contained in the α-syn variant (Fig. 1 and Supplementary Table S1). As expected, the quadruple Y-to-A mutant of α-syn devoid of tyrosines did not show tyrosine or DiY fluorescence (Supplementary Fig. S1 and Supplementary Table S1). For all tyrosine-containing variants, the occurrence of DiY fluorescence was accompanied by a decrease in tyrosine fluorescence in the range of 17%–28%, suggesting that only ~ 20% of tyrosines are converted to DiY after UV irradiation (Supplementary Fig. S1 and Supplementary Table S1). A limited yield of DiY formation upon UV irradiation has been observed before, with ~ 6% conversion to DiY in the case of calmodulin, and has been attributed to competing reactions [26].

In the case of WT α-syn, containing four tyrosines, a strong increase in DiY fluorescence was observed directly from the beginning of UV irradiation (Fig. 1b), with kinetics in agreement with a one-step first-order reaction (Fig. 1a). In contrast, all other variants, containing three or less tyrosines, showed a sigmoidal profile of DiY formation and could be fit to the reaction scheme of a consecutive two-step reaction (Fig. 1a; rate constants are given in Supplementary Table S2). The difference in the reaction profile of WT and mutant α-syn indicates that WT α-syn is exceptionally susceptible to DiY formation. The present data do not report on the step in the DiY formation mechanism responsible for this effect. However, the clear difference between WT α-syn and the single Y-to-A mutants suggests that its four tyrosines concertedly prime WT α-syn for DiY formation.

**Fig. 2:**
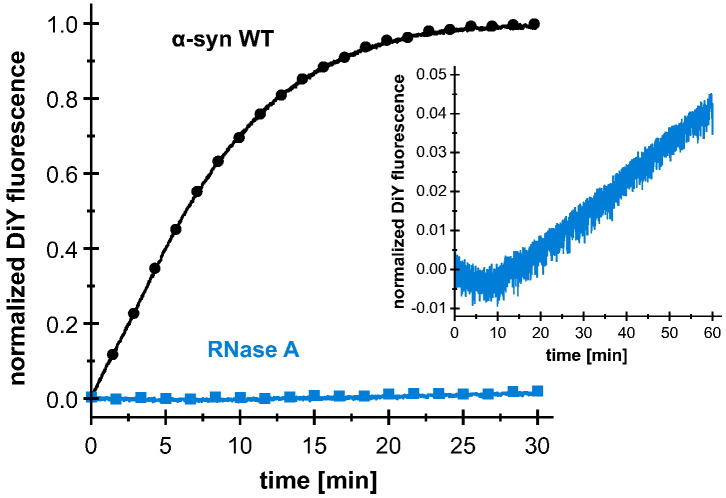
DiY formation kinetics of α-syn WT and ribonuclease A. DiY formation kinetics of 100 μM α-syn WT and 100 μM ribonuclease A (RNase A). A zoom into the DiY formation kinetics of RNase A for a longer time (1 h) is also shown. The mean curve of triplicate measurements was normalized by setting the highest fluorescence intensity of α-syn WT to unity.

One factor that may promote rapid DiY formation in α-syn is the structural flexibility of the IDP, which enables contacts between all tyrosines [33]. Ribonuclease A is a globular protein containing six tyrosine and no tryptophan residues, with the capacity to form DiY-linked dimers [47]. When UV irradiated at the same conditions, DiY formation is much slower in ribonuclease A compared to α-syn (Fig. 2), supporting a role of α-syn's conformational flexibility in facilitating DiY formation.

To evaluate the ratio of inter- to intramolecular DiY formation, dimers and monomers of UV-irradiated WT α-syn were purified by SEC (Fig. 1f). The dimer fraction contained only ~ 5% of the total protein according to absorbance at 220 nm, yet exhibited nearly the same DiY fluorescence intensity as the monomer fraction (Fig. 1g). The lower DiY content per protein in the monomer fraction is in agreement with the limited conversion of tyrosines to DiY. UV irradiation-induced α-syn dimers could also be visualized by SDS-PAGE for all α-syn variants apart from the quadruple Y-to-A mutant of α-syn devoid of tyrosines (Fig. 1e).

### DiY-modified monomers and dimers inhibit fibril formation of unmodified α-syn by interfering with fibril elongation

**Fig. 3:**
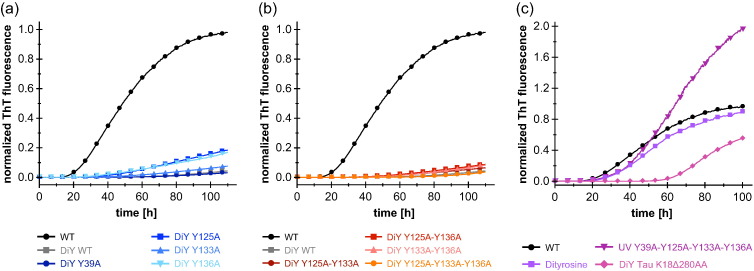
Influence of DiY-α-syn and other DiY species on the aggregation of α-syn. Kinetics of fibril formation of 25 μM non-irradiated WT α-syn in the presence of (black) 25 μM of non-irradiated WT α-syn, or in the presence of 25 μM of the indicated DiY species: (a) single Y-to-A mutants, (b) double/triple Y-to-A mutants, (c) quadruple tyrosine knockout mutant, DiY/tyrosine mix (prepared as DiY α-syn), and DiY-TauK18Δ280AA. Mean curves of triplicates are shown (with the exception of the black curve, which is the mean of 15 experiments), normalized by setting the highest fluorescence intensity in the aggregation reaction of non-irradiated WT α-syn to unity.

We next evaluated the effect of DiY crosslinks on α-syn aggregation. To this end, UV-irradiated solutions of WT and Y-to-A mutant α-syn that had achieved the final plateau of DiY fluorescence intensity (referred to hereafter as DiY-α-syn) were added to non-UV-irradiated WT α-syn, and aggregation was monitored by thioflavin T (ThT) fluorescence. The solutions contained 25 μM WT α-syn and 25 μM DiY-α-syn (total protein concentration; DiY-containing protein molecules were not further enriched). WT and Y-to-A mutant DiY-α-syn greatly inhibited aggregation of WT α-syn, manifested in a prolonged lag time and a reduced slope of the aggregation time course in the observed growth phase (Fig. 3a, b). SEC confirmed the ThT data, indicating that non-UV-irradiated WT α-syn remained largely monomeric in the presence of DiY-α-syn for a prolonged time (Supplementary Fig. S3). WT DiY-α-syn and all tyrosine-containing Y-to-A mutant DiY-α-syn variants inhibited aggregation to a similar extent. In contrast, the UV-irradiated quadruple Y-to-A mutant devoid of tyrosines (Fig. 3c) and the non-irradiated α-syn variants (Supplementary Fig. S4) did not inhibit α-syn aggregation. To investigate the specificity of aggregation inhibition by DiY-α-syn, we evaluated the inhibitory potential of free DiY and of a UV-irradiated, DiY-containing fragment of the tau protein, TauK18Δ280AA [48]. Like α-syn, tau is an IDP involved in neurodegenerative diseases, and interactions of α-syn and tau have been reported [49]. Free DiY did not show a significant effect on α-syn aggregation (Fig. 3c). DiY-TauK18Δ280AA led to a prolonged lag time of α-syn aggregation but did not have a strong effect on the slope of the aggregation time course in the observed growth phase (Fig. 3c). These observations demonstrate that the specific protein environment of DiY determines the effect on aggregation, and that DiY-α-syn is particularly potent at inhibiting α-syn aggregation.

**Fig. 4:**
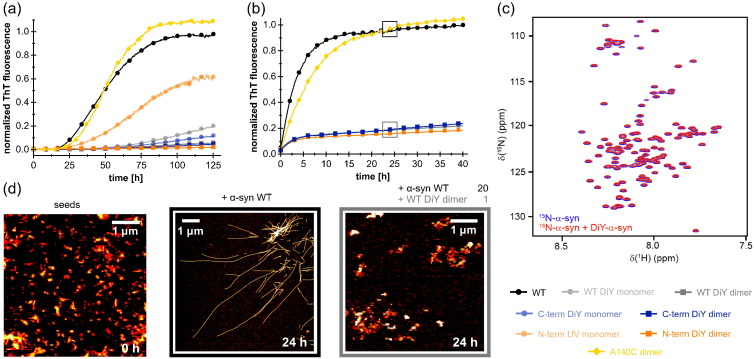
Influence of DiY-α-syn dimers and monomers on α-syn aggregation. (a) Kinetics of fibril formation of 25 μM of non-irradiated WT α-syn in the presence of either 1.25 μM DiY-α-syn dimers (WT, C-term, N-term), 1.25 μM A140C dimers, or 10 μM DiY-α-syn monomers (WT, C-term, N-term). (b) Seeded aggregation of 25 μM WT α-syn in the presence of 1.25 μM DiY dimers (WT, C-term, N-term) or 1.25 μM A140C dimers and 10% (in monomer equivalents, 2.5 μM) sonicated preformed α-syn fibril seeds. Seeded aggregation was performed without agitation to disfavor formation of new nuclei or fibril fragmentation. (c) (^1^H–^15^N) heteronuclear single quantum coherence NMR spectra of 100 μM [U–^15^N]-α-syn in the absence and presence of 400 μM unlabeled (natural abundance) WT DiY-α-syn. (d) AFM micrographs of samples taken from the seeded aggregation assay shown in panel b with/without WT DiY-α-syn dimers after 0 and 24 h.

The efficient aggregation inhibition entailed by the triple mutant Y125A–Y133A–Y136A, which can only form intermolecular DiY crosslinks through its sole tyrosine residue Y39, suggests that DiY-crosslinked α-syn dimers are the main inhibitory species (Fig. 3b). To compare the inhibitory potential of DiY-α-syn dimers and monomers, the dimeric and monomeric DiY-α-syn fractions were separated by SEC (Fig. 1f) and added to WT α-syn aggregation reactions (Fig. 4a, b). Apart from WT DiY-α-syn, the single Y-to-A mutant Y39A DiY-α-syn, which can form inter- and intramolecular DiY only in the C-terminal domain (thus denoted C-term DiY-α-syn), and the triple Y-to-A mutant Y125A–Y133A–Y136A, which can only form intermolecular DiY in the N-terminal domain (thus denoted N-term DiY-α-syn), were included in this experiment. DiY-α-syn dimers were added at a concentration of 1.25 μM to 25 μM WT α-syn, that is, at a substoichiometric ratio of 1:20 or 1:10 with regard to the dimer concentration or subunit concentration, respectively. To achieve the same DiY concentration in the case of DiY-α-syn monomers, they were added at a total protein concentration of 10 μM, accounting for the approximately eight-times lower DiY fluorescence compared to DiY-α-syn dimers (Fig. 1g). Addition of the N-term DiY-α-syn monomer fraction resulted in only minor effects on WT α-syn aggregation, in agreement with the fact that this construct cannot form intramolecular DiY crosslinks. In contrast, all DiY-α-syn dimers as well as WT and C-term DiY-α-syn monomers strongly inhibited WT α-syn aggregation (Fig. 4a). Dimers, irrespective of the domain location of the DiY crosslinks, achieved a higher inhibitory effect than monomers, supporting a dominant role of dimers in DiY-α-syn-associated aggregation inhibition.

To test the effect of DiY-α-syn on the elongation of α-syn fibrils, WT α-syn aggregation was monitored in the presence of ultrasonicated α-syn WT fibrils seeds, under conditions disfavoring formation of new nuclei or fibril fragmentation [50]. As expected under these conditions, WT α-syn aggregation proceeded rapidly without a discernible lag phase (Fig. 4b). In the presence of DiY-α-syn dimers at a substoichiometric ratio of 1:20, however, this process is halted, resulting in a ~ 5-fold lower final ThT fluorescence compared to aggregation reactions in the absence of DiY-α-syn dimers (Fig. 4b). The inhibitory effect of DiY-α-syn on fibril elongation was confirmed by atomic force microscopy (AFM). Sonicated seed particles were imaged as bundles of short (length < 200 nm) fibrils of WT α-syn [51] (Fig. 4d). After quiescent incubation of seeds with WT α-syn monomers in the absence of WT DiY-α-syn dimers, long fibrils formed (Fig. 4d). In contrast, after quiescent incubation of seeds with WT α-syn monomers in the presence of WT DiY-α-syn dimers, the seed fibril bundles appeared unchanged and long fibrils could not be observed (Fig. 4d).

We investigated if the inhibitory effect of DiY-α-syn dimers depends on the nature of the crosslink, by comparing the effect of α-syn dimers crosslinked through a C-terminal disulfide bond. Dimers of α-syn A140C had only minor effects on *de novo* and seeded aggregation of WT α-syn (Fig. 4a, b). This demonstrates that the DiY crosslink exerts specific effects.

The substoichiometric inhibition entailed by DiY-α-syn indicates that it interferes with nucleation and/or elongation of α-syn fibrils by interacting with higher-order α-syn assemblies such as oligomers, fibril surfaces, or fibril ends. We tested if DiY-α-syn also has an effect on the conformation of monomeric α-syn. For this purpose, the (^1^H–^15^N) heteronuclear single quantum coherence NMR spectra of [U–^15^N]-α-syn in the absence and presence of a 4-fold excess of [NA]-DiY-α-syn were compared (Fig. 4c). The spectra perfectly superimposed, suggesting the absence of an effect of DiY-α-syn on α-syn monomer conformation.

### DiY formation stabilizes on-pathway aggregation seeds

**Fig. 5:**
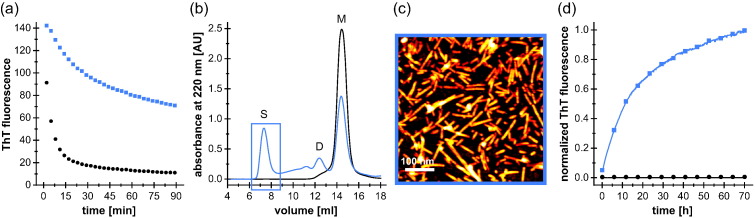
DiY crosslinking stabilizes α-syn fibril seeds. (a) Chemical denaturation in 4 M GdnHCl of WT α-syn fibrils that were (blue) or were not (black) subjected to DiY crosslinking by UV irradiation at 280 nm before, monitored by ThT fluorescence. (b) SEC after 90 min of denaturation, performed on a Superdex 200 column. S: void volume peak containing seeds (> 600 kDa); D: DiY dimer peak; M: α-syn monomer peak. The void volume fractions collected for further characterization are indicated by a blue box. (c) AFM micrograph (height image) of purified DiY-stabilized fibril seeds. (d) Seeding capacity of DiY-stabilized fibril fragments (concentration of 3.5 μM, calculated in monomer units) evaluated by addition to a fibril formation assay of 25 μM non-irradiated α-syn monomer under conditions disfavoring formation of new nuclei or fibril fragmentation (blue). The corresponding SEC fractions of denatured non-crosslinked fibrils (see panel b) were applied as a control (black).

OS-associated DiY crosslinking can stabilize preformed aggregates [33], [36], [37], [39]. We evaluated the stabilization of preformed α-syn fibrils by UV-induced DiY crosslinking against chemical denaturation and examined if DiY-stabilized α-syn aggregates retain the ability to seed α-syn fibrillation. To this end, we preformed α-syn fibrils and split the fibril sample in two halves, one of which was UV irradiated for DiY crosslinking. The samples were subsequently incubated in 4 M guanidinium chloride (GdnHCl), a potent denaturant of non-crosslinked α-syn fibrils [52]. The non-irradiated sample experienced a rapid decline in ThT fluorescence under these conditions (Fig. 5a) and eluted in SEC almost exclusively as a monomer after 90 min of incubation (Fig. 5b). In contrast, the decrease in ThT fluorescence was less pronounced for the DiY-crosslinked sample, which eluted in SEC as a mixture of monomers, low-n oligomers, and high-molecular-weight aggregates of > 600 kDa (Fig. 5a, b). AFM imaging of the void volume fraction of 4 M GdnHCl-treated DiY-crosslinked fibrils revealed small fibril fragments of about 30- to 200-nm length (Fig. 5c). These stabilized fibril fragments were subsequently added to an aggregation reaction of monomeric α-syn under conditions disfavoring formation of new nuclei or fibril fragmentation, demonstrating seeding activity of DiY-stabilized α-syn fibrils (Fig. 5d). The void volume fraction of 4 M GdnHCl-treated non-crosslinked fibrils, on the other hand, did not seed α-syn fibrillation (Fig. 5d), in agreement with the absence of stabilized fragments in this case (Fig. 5b). In conclusion, OS-associated DiY crosslinking in α-syn fibrils leads to a substantial increase in resistance to denaturation. When DiY-crosslinked fibrils are partially denatured and subsequently separated again from the denaturant, they still exhibit a strong seeding efficiency for α-syn monomers.

## Discussion

Several post-translational modifications of α-syn have been identified in Lewy bodies, potentially with critical impact on α-syn aggregation [53]. One of these modifications is DiY formation, which is special in its capacity to establish novel covalent bonds between sites that are distant in the primary sequence, as well as between two protein molecules. In the case of IDPs like α-syn, these crosslinks will strongly influence the ensemble of conformations adopted by the polypeptide chain [36]. We find that α-syn is highly susceptible to UV-induced DiY formation. This is likely another consequence of its IDP nature [33], as the globular protein ribonuclease A, which like α-syn contains tyrosines and no tryptophan, shows insignificant levels of DiY formation in comparison (Fig. 2). This is in accordance with studies on the IDP β-casein, which has a dramatically increased propensity for DiY formation compared to the globular proteins BSA and β-lactoglobulin [54]. Strikingly, WT α-syn containing four tyrosines exhibits a different time course of DiY formation from that of all mutants containing three or less tyrosines, achieving a far higher initial rate of DiY production (Fig. 1b). The very significant difference in rates cannot simply be explained by statistical arguments. Assuming that the rate of production of the tyrosine radical and its lifetime are not the limiting factors of DiY formation, but rather the encounter of two tyrosine radicals, we would expect a ratio of the rates of 6 to 3 to 1, corresponding to the number of possible intramolecular DiY pairs if a total of four, three, and two tyrosines are present per molecule of α-syn. In addition, the WT sequence with four tyrosines can in principle form two DiY crosslinks, so that the upper bound of the ratios of the intramolecular DiY formation kinetics of a purely statistical treatment is 12 to 3 to 1. Therefore, the much larger difference in response to irradiation between the WT sequence and the tyrosine deletion mutants is most likely due to differences in the efficiency in radical generation, radical lifetime, and the rates of potential side reactions other than DiY formation that the tyrosyl radicals can undergo. The distinct changes from the four-tyrosine-containing WT to the three-tyrosine-containing mutants suggest significant differences in the collective photophysical properties of the tyrosines in these constructs. These properties are likely decisively affected by the high local concentration of tyrosine in α-syn (60 mM, assuming a radius of 3 nm for the α-syn molecule) [55], and by the particular capability of tyrosine residues to establish intra- and intermolecular contacts of IDPs [56], [57].

Analysis of the Y-to-A mutants furthermore indicates that the propensity for UV-induced DiY formation does not differ greatly between the four tyrosines. This is reminiscent of a study on peroxynitrite-induced DiY formation, which arrived at the same conclusion by analyzing a set of tyrosine-to-phenylalanine mutants [34]. In contrast, other studies have observed dominant roles of Tyr-125 [35] or Tyr-133/Tyr-136 [36], or have prevalently detected Tyr-39 in DiY crosslinks [38], [39], suggesting that the method of tyrosyl radical formation affects the residue-specific DiY formation propensity.

**Fig. 6:**
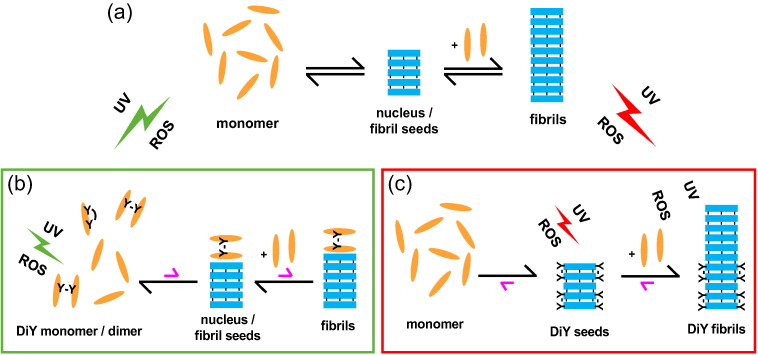
Scheme of the effects of DiY formation on α-syn aggregation. (a) Pathogenic aggregation process of α-syn from monomers to nuclei/fibril seeds and mature fibrils. (b) Inhibition of α-syn aggregation. When α-syn monomers are DiY modified by UV irradiation or ROS, resulting in DiY-crosslinked dimers and monomers, both nucleation and fibril elongation are inhibited. (c) Stabilization of α-syn fibril seeds by DiY formation. When α-syn nuclei/fibrils are modified by DiY crosslinking, they gain increased resistance to denaturation and retain the capacity to seed fibril growth.

We observe opposing effects of DiY formation on α-syn fibrillation, depending on whether monomers or fibrils are DiY modified (Fig. 6). UV-induced DiY-modification of α-syn monomers led to intra- and intermolecular DiY crosslinks, with similar amounts of DiY in the monomer and dimer fractions, but insignificant formation of higher-n oligomers (Fig. 1e, f, g). Substoichiometric amounts of DiY-modified monomers and dimers inhibited *de novo* fibril formation as well as seeded fibril growth of unmodified α-syn (Fig. 6b). This demonstrates that at least the fibril elongation step of the aggregation reaction is inhibited. Since free DiY and a DiY-containing fragment of the tau protein showed no or only weak effects on α-syn fibrillation, respectively, specific interactions between DiY-modified and -unmodified α-syn molecules seem to be critical for aggregation inhibition. The hydrophobic NAC region might be a key interaction site in this context, as it is critical for α-syn self-assembly. At the same time, the inhibitory effect is not a general phenomenon linked only to dimer formation, as a dimer linkage through a C-terminal disulfide bond could not reproduce the effect of C-terminal DiY crosslinking (Fig. 4a, b). This suggests that DiY is not just serving as a linker that modulates the conformational ensemble of α-syn, but that the DiY group is actively involved in interactions that lead to an inhibition of fibril elongation. The position of the crosslinked tyrosines within the primary sequence does not seem to be critical for these inhibitory interactions, since they are established by both purely N-terminal crosslinks (via Tyr-39, which is part of the β-sheet core in the fibrillar state [58], [59], [60], [61], [62], [63]) and purely C-terminal crosslinks (via Tyr-125, Tyr-133, or Tyr-136, within the charged tail that remains disordered in the fibrillar state). The substoichiometric inhibition of fibril elongation suggests that these interactions occur preferentially at the fibril end. With fibril ends, DiY-modified monomers and dimers establish interactions that are incompatible with fibril elongation. The stronger inhibitory effect of the dimer compared to the monomer could be due to the larger number of sites in the dimer that are involved in such off-pathway interactions (e.g., two NAC regions in the dimer *versus* one in the monomer), or due to the increased topological challenge to correctly incorporate a dimer into the in-register fibril core. We note again, however, that the specific DiY crosslink is critical for the enhanced inhibition of the dimer, as a dimer linkage through a C-terminal disulfide bond did not show a significant inhibitory effect.

Previous studies have shown that DiY crosslinking stabilizes preformed α-syn aggregates against chemical denaturation [33], [36], [37], [39]. We find that preformed α-syn fibrils stabilized by UV-induced DiY modification are effective at seeding α-syn fibril growth (Figs. 5d and 6c). Stabilization of α-syn fibril seeds by DiY crosslinking might consequently be a factor that counteracts their cellular degradation and hence supports the persistence and even spreading of α-syn pathology. Which tyrosine residue(s) in α-syn might be responsible for this effect? Several studies on the structure of α-syn fibrils have shown that Tyr-39 is part of the parallel, in-register β-sheet core [58], [59], [60], [61], [62], [63]. Tyr-39 is not located within the Greek key motif [63], but in a peripheral β-sheet. The addition of an intermolecular covalent bond to this region between adjacent Tyr-39 side chains might well lead to increased fibril stability. Moreover, the C-terminal tyrosines Tyr-125, Tyr-133, and Tyr-136 of different monomers are also in proximity to each other within the disordered tails at the fibril surface and thus available for crosslinking, which could further contribute to fibril stability.

In contrast to the consistent observation of stabilization of preformed α-syn aggregates [36], previous studies reported different effects of DiY crosslinking on *de novo* fibril formation. In agreement with the inhibitory effect of DiY-modified α-syn monomers and dimers reported here, Norris *et al*. [34] found that α-syn did not polymerize into fibrils after peroxynitrite treatment. Tris(bipyridine)ruthenium(II) chloride photosensitization led to a reduction in fibril yield [39]. In contrast, formation of off-pathway aggregates or increased fibril formation was observed upon treatment with cytochrome c/H_2_O_2_ [36] or CuCl_2_/H_2_O_2_ [30], respectively. Specifically for DiY-crosslinked α-syn dimers, a reducing effect on the fibril yield of non-crosslinked α-syn [39] or a role as critical on-pathway intermediate [37] has been reported. The disparate findings suggest that the conditions of tyrosyl radical generation modulate the effects of DiY-α-syn on fibril formation, for example, by determining the extent of DiY modification and the set of DiY-modified α-syn species. In fact, we observe exclusively DiY-modified α-syn monomers and dimers after UV irradiation, whereas previous protocols generated α-syn multimers up to high-n oligomers [30], [33], [34], [36], [39].

We find that DiY-modified monomers and dimers strongly inhibit fibril elongation, whereas DiY stabilization of fibril seeds promotes fibril elongation in the presence of a degradation/destabilization process. DiY modification of α-syn might therefore protect from aggregation in the absence of aggregates, or when low aggregate amounts are present. However, as aggregation proceeds in the course of disease progression, DiY formation might switch from an aggregation-inhibiting to an aggregation-promoting factor above a critical level of aggregate formation. In the presence of secondary nucleation processes that lead to autocatalytic proliferation of fibrils [50], the rate of which depends on the total mass of fibrils, DiY-induced stabilization of fibrils could ultimately result in the initiation of a positive feedback loop of α-syn aggregation.

## Materials and Methods

### Expression and purification of acetyl-α-syn and acetyl-α-syn mutants

Acetyl-α-syn WT, acetyl-α-syn Y-to-A mutants and acetyl-α-syn-A140C were expressed in *Escherichia coli* BL21DE3 carrying codon-optimized α-syn in pT7–7 vector and the pNatB vector with the N-terminal acetylation enzyme NatB from *Schizosaccharomyces pombe* [64]. PNatB (pACYCduet-naa20-naa25) was a gift from Dan Mulvihill (Addgene plasmid no. 53613). Y-to-A mutants were generated by QuikChange site-directed mutagenesis. Expression was conducted in phosphate-buffered (50 mM, pH 7.2) 2YT-medium with 0.4% glycerol and 2 mM MgCl_2_; protein production was induced at ~ OD 1.2 with 1 mM IPTG and run for 4 h at 37 °C. Purification of α-syn was performed as previously described [65] with modifications to the original protocol: Cell lysis and release of thermostable α-syn was carried out by boiling the frozen cell pellet in a fourfold volume of dH_2_O at 95 °C for 30 min. After centrifugation at 15,000*g* and 4 °C for 30 min, the protein in the supernatant was precipitated by gradually adding saturated ammonium sulfate solution until 50% saturation was reached. Protein was pelleted at 15,000*g* and 4 °C for 30 min and resuspended in 50 ml of 50 mM Tris–HCl at pH 8, sterile-filtered, and loaded onto a 5-ml HiTrap Q FF anion exchange chromatography column (GE Healthcare). Acetyl-α-syn eluted at ~ 300 mM NaCl in a 0- to 500-mM NaCl gradient. The elution fractions were precipitated by adding saturated ammonium sulfate solution to 50% saturation, the protein pelleted as before, resuspended in dH_2_O and finally purified by SEC on a Superdex 75 16/60 column (GE Healthcare) in 25 mM K-phosphate buffer at pH 7.3 and 100 mM KCl. Final yields were ~ 40–60 mg/L culture for acetyl-α-syn WT and Y-to-A mutants, and around 20 mg/L for acetyl-α-syn A140C. Protein concentrations were determined by absorbance measurement at 275 nm using an extinction coefficient of 5600 M^− 1^ cm^− 1^ if not stated otherwise.

### DiY formation by UV irradiation

For DiY formation, 100 μM solutions of α-syn WT and α-syn Y-to-A mutants as well as tauK18Δ280AA, tyrosine, and ribonuclease A in 25 mM K-phosphate buffer at pH 7.3 and 100 mM KCl were loaded into a fluorescence cuvette with a magnetic stir bar. All spectra and DiY formation kinetics were recorded on a JASCO FP-6500 spectrofluorometer at 20 °C. Before and after DiY formation, tyrosine spectra (*λ*_Ex_ = 274 nm, bandwidth 5 nm; *λ*_Em_ = 290–350 nm) and DiY spectra (*λ*_Ex_ = 320 nm, bandwidth 5 nm; *λ*_Em_ = 350–480 nm) were recorded. DiY formation was induced at *λ*_Ex_ = 280 nm (bandwidth 20 nm) and monitored every second at *λ*_Em_ = 410 nm (bandwidth 20 nm). UV irradiation and fluorescence readings were stopped when a fluorescence intensity plateau was reached to prevent excessive photobleaching. For evaluation, buffer fluorescence was subtracted and the mean of triplicate measurements was normalized by setting the highest fluorescence of WT DiY-α-syn to unity. The kinetics of DiY formation were fit to a consecutive two-step reaction or a one-step first-order reaction, using Abscissa 2D plot and fit tool by Rüdiger Brühl†.

### Separation of DiY-α-syn dimers and DiY-α-syn monomers

To compare the inhibitory potential of DiY-α-syn dimers and monomers, the dimeric and monomeric fractions of WT DiY-α-syn, the single Y-to-A mutant Y39A DiY-α-syn (C-term DiY-α-syn), and the triple Y-to-A mutant Y125A–Y133A–Y136A (N-term DiY-α-syn) were separated by SEC. DiY formation was carried out in solutions of 200 μM WT α-syn, 267 μM Y39A α-syn, and 800 μM Y125A–Y133A–Y136A α-syn, that is, at equivalent tyrosine concentration. After DiY formation by UV irradiation, 500 μl of each sample was loaded onto a Superdex 75 10/300 column (GE Healthcare) and separated by SEC in 25 mM K-phosphate buffer at pH 7.3 and 100 mM KCl. For analytical SEC, 200-μl samples with half the protein concentration were loaded. As DiY formation significantly changed the absorption of α-syn at 275 nm and to ensure comparability of the different Y-to-A mutants, protein concentrations were measured at 220 nm using extinction coefficients of 105,344 M^− 1^ cm^− 1^ for monomers and 210,688 M^− 1^ cm^− 1^ for dimers. These extinction coefficients were determined by measuring the absorbance at 220 nm of a WT α-syn sample of known concentration (determined by absorbance at 275 nm). For determination of the dimer and monomer yields, SEC peaks were integrated by the UNICORN evaluation software (GE Healthcare). For the measurement of DiY fluorescence in the dimer and monomer peaks, three ~ 400-μl samples of 100 μM WT DiY-α-syn were separated by SEC, the protein concentrations of dimer and monomer peaks were measured as before, and the fluorescence was measured at 410 nm with *λ*_Ex_ = 320 nm. The mean DiY fluorescence of triplicate samples was calculated.

### Aggregation assays

ThT aggregation assays were conducted in Corning half area 96-well plates with non-binding surface (Corning No. 3881). For the assays starting from monomeric α-syn, 25 μM of non-irradiated WT α-syn was mixed with either 25 μM DiY-α-syn, 25 μM of UV-irradiated tyrosine, or 25 μM of UV-irradiated tauK18Δ280AA. Aggregation assays were run for 5 days with measurement of ThT fluorescence every 20 min (*λ*_Ex_ = 450 nm, bandwidth 5 nm; *λ*_Em_ = 482 nm, bandwidth 10 nm) with 15 s of orbital shaking before the measurement in Tecan Infinite 200PRO and Tecan Infinite M1000PRO plate readers. The assays were conducted at 37 °C in 25 mM K-phosphate buffer at pH 7.3, 100 mM KCl, 1 mM MgCl_2_, 10 μM ThT, and 0.05% NaN_3_, reflecting intracellular potassium and magnesium concentration as well as intracellular pH and ionic strength. A glass ball was added per well to improve mixing. Per well, 125-μl sample was used. To evaluate the effect of DiY-α-syn dimers and A140C dimers on α-syn aggregation, 1.25 μM dimers (WT DiY-α-syn dimer, C-terminal DiY-α-syn dimer, N-terminal DiY-α-syn dimer, A140C dimer) were added to 25 μM of WT monomers (1:20 stoichiometry). DiY-α-syn monomer fractions were used at a total α-syn concentration of 10 μM in order to achieve the same DiY concentration as in the dimer experiments, considering the eight-times lower DiY fluorescence compared to DiY-α-syn dimers. For seeded aggregation assays, 10% (in monomer units, 2.5 μM) of WT α-syn fibril seeds were added to 25 μM WT α-syn aggregation reactions. The seed solution was prepared as follows: 300 μl of 100 μM α-syn was fibrillated at 37 °C and 800 rpm for 3 days in a 2-ml tube containing a glass balls in a Thermomixer (Eppendorf). The fibril solution was diluted to 50 μM and sonicated with a tip sonicator (Bandelin Sonopuls HD3200, BANDELIN electronic) at 10% power (20 W) for 60 s, with 1-s pulses separated by 4-s pause. In seeded aggregation experiments, samples were not shaken prior to measurement to disfavor formation of new nuclei or fibril fragmentation. For evaluation, the mean of triplicate measurements was referenced to the highest fluorescence of 25 μM WT α-syn.

### NMR spectroscopy

NMR spectra were acquired at 10 °C using a 750-MHz Avance III spectrometer (Bruker) equipped with a cryogenically cooled Z-axis pulse-field-gradient triple-resonance probe. The NMR samples contained [U–^15^N]-α-syn at a concentration of 100 μM in 20 mM Na-phosphate buffer at pH 7.4, 50 mM NaCl, and 8% D_2_O, with or without the addition of 400 μM [NA]-DiY-α-syn.

### AFM

For AFM imaging, 20 μl of the indicated samples shown in Fig. 4, Fig. 5 was incubated for 30 min on a gold-coated mica surface; the solution was taken off, shortly washed with dH_2_O, and dried under a nitrogen stream. The samples were imaged by tapping mode on a JPK Instruments Nanowizard 3.

### Denaturation and seeding capacity of DiY-crosslinked α-syn fibrils

For the fibril denaturation experiments, 3 ml of 150 μM α-syn WT in 25 mM K-phosphate buffer at pH 7.3, 100 mM KCl, 1 mM MgCl_2_, and 0.1% NaN_3_ was fibrillated at 37 °C in a 15-ml tube under stirring with a magnetic stir bar for 3 days. Four aliquots of 700 μl were directly frozen in liquid nitrogen and stored at − 80 °C. One-half of each aliquot was subjected to DiY crosslinking by UV irradiation as described before. Subsequently, 300 μl of the fibril solutions (with and without DiY crosslinks) was mixed with 20 μM ThT and 4 M guanidinium chloride at pH 8 in a glass cuvette with a stir bar (total volume: 600 μl) and incubated for 90 min at 20 °C inside the fluorometer. ThT fluorescence was measured every min at 485 nm (*λ*_Ex_ = 445 nm). Buffer fluorescence was subtracted. After 90 min of denaturation, 500 μl of the solutions was loaded onto a Superdex 200 10/300 column (GE Healthcare) and separated at a flow rate of 0.5 ml/min in 25 mM K-phosphate buffer at pH 7.3 and 100 mM KCl. Void volume fractions (~ 1.5 ml) were collected and directly evaluated for their capacity to seed fibril formation by the addition (80% v/v) to aggregation reactions of 25 μM non-irradiated WT α-syn at 37 °C. Fibril formation was followed without shaking to disfavor formation of new nuclei or fibril fragmentation.

## Appendix A Supplementary data

Supplementary material Image 2

## Footnotes

† http://rbruehl.macbay.de/
