# Supplementary material for: Opposed Effects of Dityrosine Formation in Soluble and Aggregated α-Synuclein on Fibril Growth
Source: J Mol Biol. 2017 Oct 13;429(20):3018–30. doi: 10.1016/j.jmb.2017.09.005 (PMC5637163; doi:10.1016/j.jmb.2017.09.005)
Supplement: Supplementary file 1 — Supplementary material [file mmc1.pdf]

Supporting Information

Opposed Effects of Dityrosine Formation in Soluble and Aggregated α-Synuclein on Fibril Growth

Michael M. Wördehoff[1], Hamed Shaykhalishahi[1], Luca Groß[1], Lothar Gremer[1,2], Matthias Stoldt[1,2], Alexander K. Buell[1], Dieter Willbold[1,2] and Wolfgang Hoyer[1,2,*]

[1] Institut für Physikalische Biologie, Heinrich-Heine-Universität Düsseldorf, 40204 Düsseldorf, Germany.
[2] Institute of Complex Systems (ICS-6), Structural Biochemistry, Forschungszentrum Jülich, 52425 Jülich, Germany.
[*] Email: wolfgang.hoyer@uni-duesseldorf.de

Contents: Supplementary Figures S1, S2, S3, S4 and Supplementary Tables S1 and S2.

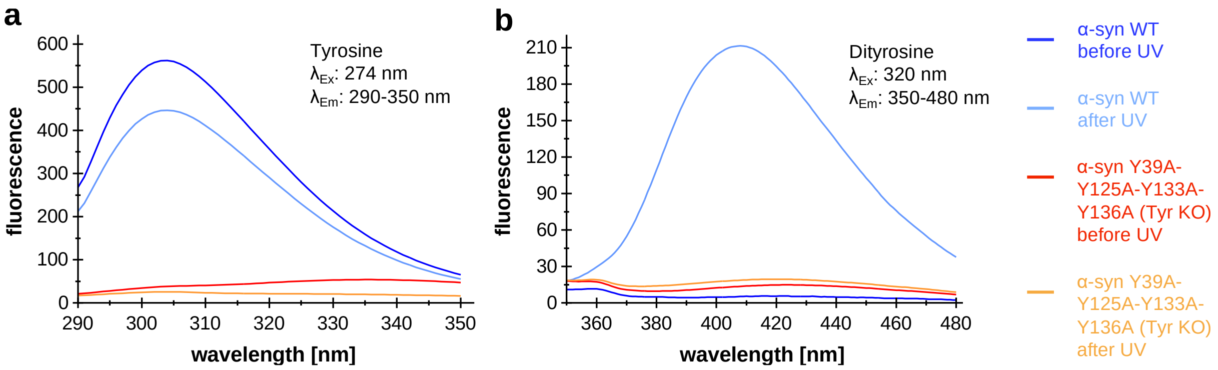

Figure S1. Tyrosine and dityrosine spectra of α-syn WT and α-syn Y39A-Y125A-Y133A-Y136A (Tyr KO) mutant before and after UV-irradiation. (**a**) Tyrosine spectra of WT and Tyr KO mutant before and after UV-irradiation recorded at λ_{Ex}: 274 nm and at λ_{Em}: 290-350 nm. Whereas α-syn WT shows a marked tyrosine spectrum with a peak at 303 nm that decreases ~20% after UV-irradiation, the Tyr KO mutant does not exhibit a marked tyrosine spectrum. (**b**) Dityrosine spectra of WT and Tyr KO mutant before and after UV-irradiation recorded at λ_{Ex}: 320 nm and at λ_{Em}: 290-350 nm. α-Syn WT shows a high DiY fluorescence increase at 410 nm after UV-irradiation, the Tyr KO mutant does not show a peak at 410 nm after UV irradiation.

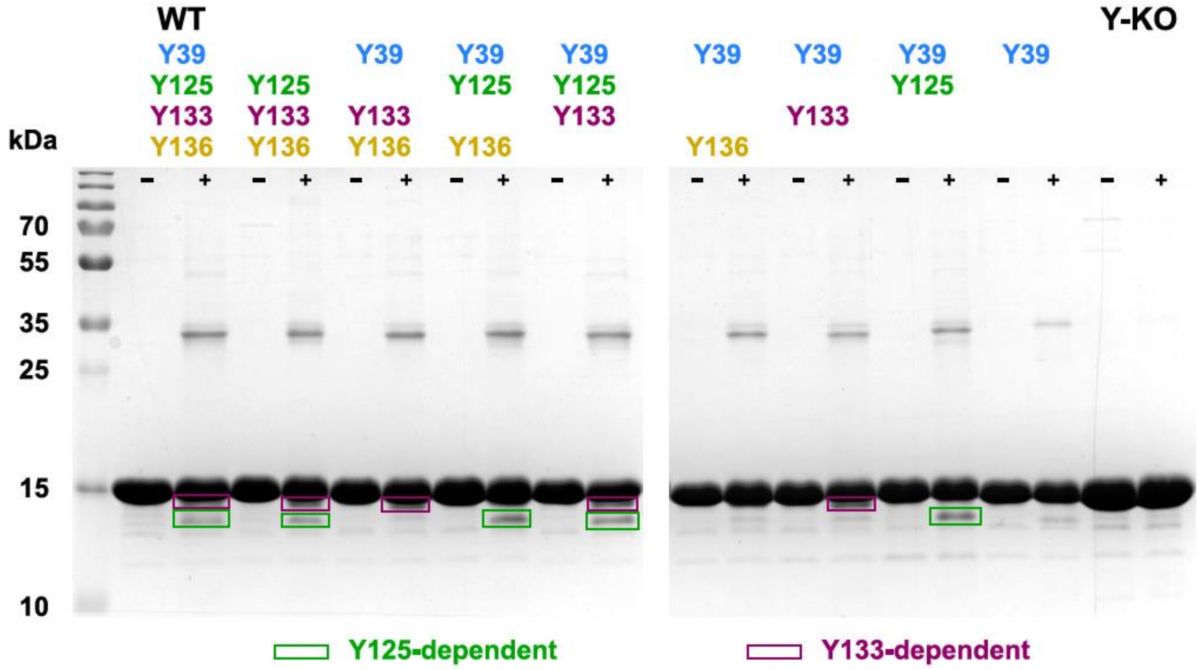

Figure S2. Low molecular weight bands in SDS-PAGE indicate tyrosine-dependent UV-induced fragmentation of α-syn. SDS-PAGE of WT α-syn and Y-to-A mutants before (-) and after (+) UV irradiation, same gels as in Fig. 1e. Upon UV-irradiation, additional bands appear below the monomer bands of the 140 amino acid monomeric proteins. The two most intense lower MW bands are only observed for constructs containing Tyr-125 (green) or Tyr-133 (purple), respectively, and are not dependent on the presence or absence of any other specific tyrosine residue. This indicates that the bands originate from tyrosine-dependent UV-induced fragmentation and do not represent full-length monomers containing specific intramolecular DiY-crosslinks with distinct migration behavior. Tyrosine-mediated backbone cleavage upon UV exposure of polypeptides has been reported before [1,2].

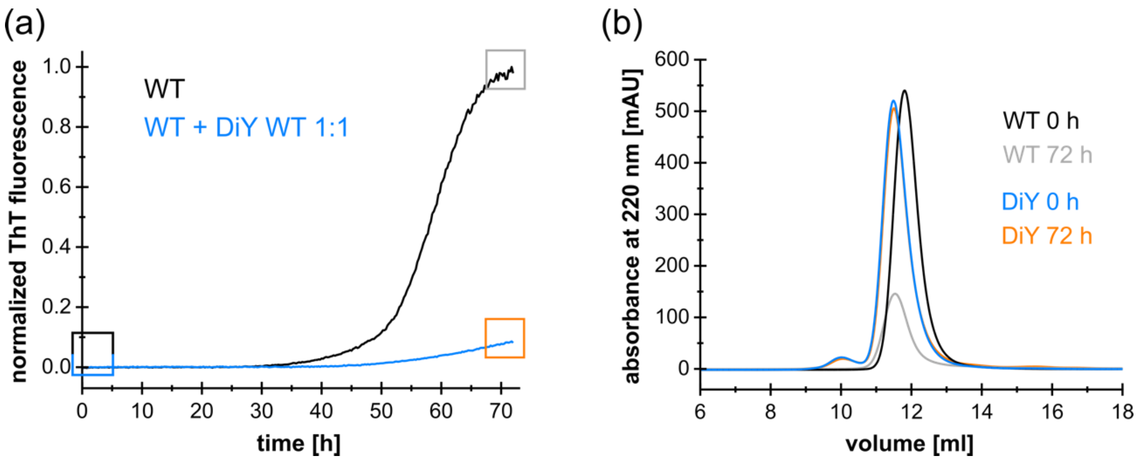

Figure S3. Analysis of aggregated samples of α-syn WT and DiY α-syn WT by size exclusion chromatography (SEC). (a) 25 µM α-syn WT was aggregated in the presence of 25 µM α-syn WT or 25 µM DiY α-syn WT and 10 µM Thioflavin T in 25 mM K-phosphate buffer pH 7.3, 100 mM KCl and 0.05% NaN_3. Mean of triplicate measurements, normalized to the highest WT fluorescence are shown. At 0 h and after 72 h of aggregation, samples were recovered from the aggregation assay, pooled and centrifuged at 20,000 *g* and RT for 30 min. Approximately 450 µl of the supernatants were loaded onto a Superdex 75 10/300 SEC column (b). SEC analysis revealed that in the case of α-syn WT, only ~30% of protein is left monomeric after 72 h of aggregation (black vs. grey curve). In the case of α-syn WT + DiY α-syn WT (1:1), nearly all protein is still monomeric (and dimeric) after 72 h of aggregation (blue vs. orange curve). No higher molecular weight oligomers could be detected.

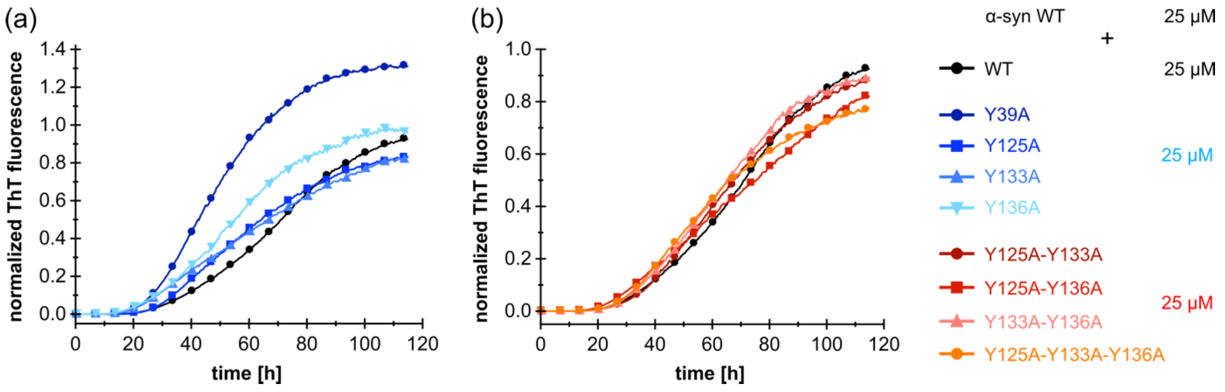

Figure S4. Aggregation of α-syn WT in presence of non-irradiated α-syn Y-to-A mutants. Analogous to the aggregation assays shown in figure 3, 25 µM α-syn WT was mixed with 25 µM of non-irradiated α-syn single Y-to-A mutants (a) and non-irradiated α-syn double/triple Y-to-A mutants (b) and aggregated for 120 hours in the presence of 10 µM Thioflavin T in 25 mM K-phosphate buffer pH 7.3, 100 mM KCl and 1 mM $MgCl_2$. Mean of triplicates normalized to the highest Thioflavin T fluorescence of α-syn WT is shown. The mean curve for α-syn WT is based on 12 measurements. Non-irradiated Y-to-A mutants did not inhibit the aggregation of α-syn WT.

Table S1. Tyrosine and DiY fluorescence emission of α-syn variants before and after DiY formation. For the quadruple mutant, tyrosine and dityrosine spectra could not be measured properly because of the absence of tyrosines, n. o.: not observed**.**

Protein	Tyrosine fluorescence λ_{Ex}: 274 nm, λ_{Em}: 303 nm			Dityrosine fluorescence λ_{Ex}: 320 nm, λ_{Em}: 410 nm	
	absolute		decrease [%]	absolute	
	before	after		before	after
WT	560	451	19.5	13	356
Y39A	376	288	23.3	6	109
Y125A	453	378	16.7	7	138
Y133A	514	410	20.2	9	136
Y136A	465	367	21.0	12	215
Y125A-Y133A	399	329	17.4	9	146
Y125A-Y136A	340	279	18.1	11	108
Y133A-Y136A	395	320	18.9	10	134
Y125A-Y133A-Y136A	243	176	27.6	5	27
Y39A-Y125A-Y133A-Y136A (Tyr KO)	n. o.	n. o.	-	n. o.	n. o.

Table S2. Rate constants of DiY formation in α-syn. The kinetics data of WT α-syn was fit to a first-order reaction, while all other data was fit to a consecutive two stage reaction $A \overset{k_1}{\rightarrow} B \overset{k_2}{\rightarrow} C$.

Protein	Rate constants	
	k_m [s^{-1}]	k_n [s^{-1}]
WT	$4.02 \cdot 10^{-3}$	-
Y39A	$4.58 \cdot 10^{-3}$	$4.58 \cdot 10^{-3}$
Y125A	$1.01 \cdot 10^{-2}$	$2.95 \cdot 10^{-3}$
Y133A	$7.75 \cdot 10^{-3}$	$3.09 \cdot 10^{-3}$
Y136A	$2.01 \cdot 10^{-2}$	$3.34 \cdot 10^{-3}$
Y125A-Y133A	$1.23 \cdot 10^{-2}$	$3.77 \cdot 10^{-3}$
Y125A-Y136A	$1.40 \cdot 10^{-2}$	$3.82 \cdot 10^{-3}$
Y133A-Y136A	$2.38 \cdot 10^{-2}$	$3.77 \cdot 10^{-3}$
Y125A-Y133A-Y136A	$5.33 \cdot 10^{-3}$	$1.33 \cdot 10^{-3}$

References

[1] D.I. Pattison, M.J. Davies, Actions of ultraviolet light on cellular structures., EXS. (2006) 131–157. doi:10.1007/3-7643-7378-4_6.

[2] A. Wright, W.A. Bubb, C.L. Hawkins, M.J. Davies, Singlet oxygen-mediated protein oxidation: evidence for the formation of reactive side chain peroxides on tyrosine residues, Photochem Photobiol. 76 (2002) 35–46. doi:10.1562/0031-8655(2002)0760035SOMPOE2.0.CO2.
